# Simulated Microgravity Using a Rotary Culture System Compromises the *In Vitro* Development of Mouse Preantral Follicles

**DOI:** 10.1371/journal.pone.0151062

**Published:** 2016-03-10

**Authors:** Shen Zhang, Dahan Zheng, Yonggen Wu, Wei Lin, Zaichong Chen, Luhe Meng, Jun Liu, Ying Zhou

**Affiliations:** 1 Reproductive Medicine Center, First Affiliated Hospital of Wenzhou Medical University, Wenzhou, Zhejiang Province, China; 2 School of Laboratory Medicine and Life Science, Wenzhou Medical University, Wenzhou, Zhejiang Province, China; 3 Stem Cells and Genetic Engineering Group, Department of Materials Engineering, Monash University, Clayton, Victoria, Australia; 4 Department of Histology and Embryology, Wenzhou Medical University, Wenzhou, Zhejiang Province, China; China Agricultural University, CHINA

## Abstract

**Background:**

Growing cells in simulated weightlessness condition might be a highly promising new technique to maintain or generate tissue constructs in a scaffold-free manner. There is limited evidence that microgravity condition may affect development of ovarian follicles. The objective of the present study was to investigate the effects of simulated microgravity on the *in vitro* development of mouse preantral follicles.

**Methods and Results:**

Ovarian tissue from 14-day-old mice, or preantral follicles mechanically isolated from 14-day-old mouse ovaries were cultured at a simulated microgravity condition generated using a rotating wall vessel apparatus. Follicle survival was assessed quantitatively using H&E staining. Follicle diameter and oocyte diameter were measured under an inverted microscope. Ultrastructure of oocytes was evaluated using transmission electron microscopy. We observed that simulated microgravity compromised follicle survival *in vitro*, downregulated PCNA and GDF-9 expressions, and caused ultrastructural abnormalities in oocytes.

**Conclusion:**

This study showed for the first time that three-dimensional culture condition generated by simulated microgravity is detrimental to the initial stage development of mouse preantral follicles *in vitro*. The experimental setup provides a model to further investigate the mechanisms involved in the *in vitro* developmental processes of oocytes/granulosa cells under the microgravity condition.

## Introduction

*In vitro* ovarian follicle culture systems have been developed and can be used to study the dynamics of oocyte-grannulosa cell interactions, hormone secretions and oocyte maturation [1]. Culture of preantral follicles on a flat surface of petri dishes has resulted in live birth in mouse [2–4]. Culture of early ovarian follicles in alginate beads (three dimensional, 3D) for the duration of follicle development to the antral stage allows maintaining natural follicular 3D structure, which may be important to keep similar gene expression patterns as *in vivo* [5] and produce meiotically competent oocytes [6, 7]. A 3D culture makes cells mimic tissue-like structure more effectively than a 2-dimensional culture. Generally, 3D culture methods can be established using scaffolds (such as hydrogel and inert matrix) and scaffold-free solutions (such as hanging drop suspension, rolling vessel or magnetic levitation). Rolling vessels that generate simulated microgravity (s-μ*g*) conditions, such as rotating wall vessel (RWV), has been developed and used for tissue engineering [8]. The RWV, preventing cells from aggregation through a constant rotation, has been developed by national Aeronautics and Space Administration (NASA) [9]. The rotation speed has to be adjusted to the specific weight of the cells/tissue, the medium density, and viscosity. The constantly falling of cells/tissues within the rotating medium in the RWV generates a simulated microgravity for the samples cultured in the vessels.

3D cell culture systems using simulated microgravity technologies, such as RWV, have provide a scaffold-free approach in tissue engineering. Human endothelial cells formed intima constructs that might be a first step to the generation of fully functional vessels [10, 11]. In addition, thyroid cells cultured at s-μ*g* condition for several days formed small spherical aggregates, which can be grown to a state to resemble thyroid tissue [12]. Further, chondrogenesis can be promoted from adipose-derived mesenchymal stem cells in an RWV bioreactor [13]. However, studies also showed that s-μ*g* compromised mouse oocyte maturation by disrupting meiotic spindle organization and inducing cytoplasmic blebbing [14]. Moreover, s-μ*g* elevated the expressions of phosphorylated stress-activated protein kinase (SAPK) and junC N-terminal kinase (JNK), that trigger stress-induced apoptosis and cell cycle arrest, in mouse preimplantation embryos [15]. In this study, ovarian tissue containing preantral follicles and isolated preantral follicles from 14-day-old mice were used to investigate the effects of s-μ*g* on development of the follicles and growth of the oocytes *in vitro*.

## Materials and Methods

### Animals

ICR mice (n = 80) used in this study were housed and bred at the SPF facility of Animal Centre of Wenzhou Medical College. Ethical approval for this study was obtained from the Animal Research Ethics Committee, Wenzhou Medical College.

### Follicle isolation and encapsulation

14-day-old mice were killed by cervical dislocation, and the ovaries were aseptically removed and collected in L-15 medium supplemented with 10% fetal bovine serum (FBS), 100 IU/ml penicillin, and 100 μg/ml streptomycin (all from Sigma-Aldrich). For ovarian tissue culture, each ovary was cut into half with approximate 1 mm in thickness. For follicle culture, follicles were mechanically isolated from ovaries using 26 1/2-guage needles (Becton Dickinson, BD) under a stereomicroscope. The preantral follicles included in the study were selected according to the following criteria: 1) round and centrally located oocytes surrounded by 2–3 layers of grannulosa cells, 2) intact basal membrane to which some theca cells were attached, 3) 90–100 μm follicle diameter.

### Encapsulation of follicles

Alginate hydrogel preparation and follicle encapsulation were performed as described elsewhere with modification [6]. Briefly, 0.8% (w/v) sodium alginate (Sigma) solution was reconstituted with sterile PBS; then 10 μl of the alginate solution was dropped into the encapsulation solution (140 mM NaCl / 50 mM CaCl_2_) to create alginate beads; then, single follicles were pipetted into the middle of each alginate bead. After rinsing in culture medium, alginate beads containing a single follicle were cultured in 35×10 mm dishes or in the vessel under simulated microgravity treatment.

### *In vitro* culture of ovarian tissue or encapsulated follicles under simulated microgravity treatment

The culture medium consisted of alpha-minimal essential medium (α-MEM) (Sigma) supplemented with 5% FBS, 1% insulin-selenium-transferrin (Invitrogen), 10 mIU/ml rFSH (Merck Serono), 100 IU/ml penicillin and 100 μg/ml streptomycin. In the experimental group, to decrease the net gravitational force on the encapsulated follicles or ovarian tissue, a Synthecon rotating wall vessel (RWV) apparatus for simulating microgravity was used. The RWW microgravity culture system was set up as described previously [15]. Briefly, the high-aspect-ratio rotating vessel (HARV) was filled with light paraffin oil (~10 ml, Vitrolfie), then 150 μl of culture medium containing one piece of ovarian tissue or three encapsulated follicles was placed into the oil inside HARV. In control group, alginate beads containing single follicles or ovarian tissue were cultured in 150 μl of culture medium covered with light paraffin oil in 35×10mm dishes (BD). Both culture dishes and RWV apparatus were incubated in a humidified incubator at 37°C with 5% CO_2_ in air.

### Follicle retrieval

Encapsulated follicles were retrieved from the alginate beads after 4 days culture. The beads were transferred to L15 medium supplemented with 10 units/ml alginate lyase (Sigma) for 30 minutes at 37°C. Follicles were removed from the degraded alginate bead and washed in L15 medium with 10% FBS.

### Cell viability assay

Follicles after decapsulation were washed three times in D-PBS. About 10 follicles were incubated in 150 μl of the combined LIVE/DEAD assay reagents (2 μM calcein AM and 4 μM EthD-1, Thermo-fisher) at room temperature for 45 min. Following incubation, follicles were transferred to a clean microscope slide with 10 μl D-PBS, covered with a coverslip and sealed with clear fingernail polish. The live and dead granulosa cells in follicles were examined under a confocal fluorescence microscope. Follicles with less than 10% dead cells are considered as healthy survived follicles.

### Ovarian tissue and follicle fixation and H&E staining

Fresh ovaries and isolated preantral follicles (day 0) were fixed after they were removed from 14-day-old mice. Cultured ovarian tissue was fixed after either 2 days or 4 days culture. Individual follicle was fixed inside the alginate bead after 4 days culture. The fixation was performed in Bouin’s solution (Sigma) at room temperature for 24 hours. All samples were embedded in paraffin, serially sectioned at 5 um and stained with hematoxylin and eosin (H&E).

### Follicle counting the measurement of follicle/oocyte diameter

In ovarian tissue sections strained by H&E, healthy preantral follicles containing at least two layers of granulosa cells were counted and follicle density was calculated by dividing the numbers of follicles with the tissue section area. Follicle and oocyte diameter of the freshly isolated and 4-day-cultured follicles were measured. Measurement of the diameters was performed with a caliper in the eyepiece of an inverted microscope. For each measurement, the mean diameter of a follicle or an oocyte was estimated by means of averaging two perpendicular diameters of the subject.

### Immunohistochemistry

For immunohistochemistry, ovarian tissue were fixed in 4% paraformaldehyde and embedded in paraffin. 5-μm-thickness sections were dewaxed, rehydrated and performed heat mediated antigen retrieval in Citrate buffer for 15 minutes. After blocking the endogenous peroxidase by 3% hydrogen peroxide and unmasking the antigen by 5% goat serum, the sections were incubated with primary antibodies for 3 hours at room temperature. After washing, sections were incubated with 1:500 diluted horseradish peroxidase (HRP)-conjugated goat anti-rabbit IgG (H+L) (111-035-003, Jackson Immuno-Research Laboratories) for 1 hour at room temperature. Sections were incubated with 3,3N-Diaminobenzidine Tertrahydrochloride (DAB, Beyotime) to detect the peroxidase activity and mounted with aqueous mounting medium (Dako). The primary antibodies were rabbit anti-GDF-9 antibody (BS-175R, Beijing Biosynthesis Biotechnology, 1:500 dilution) and rabbit anti-PCNA antibody (24036-1-AP, Proteintech, 1:200 dilution).

### Transmission electron Microscopy

Follicles were fixed in 2.5% glutaraldehyde for 3 hours at room temperature, then post-fixed in 1% osmium tetroxide for 1 hour at 37°C. After washing with PBS, the follicles were treated with 1% phosphotungstic acid and 1% sodium uranyl acetate for 1 hour at 37°C, and dehydrated through acetone series and epoxy resin-acetone mixture at 37°C, then embedded in epoxy resin at 45°C. Semi-thin sections were cut and stained with methylene blue (Sigma) to localize the follicles in the blocs. Ultrathin sections were cut and mounted on grids, and evaluated with H-7500 transmission electron microscope (Hitachi).

### Statistics

All data were presented as mean ± SEM (standard error of the mean). Prior to the statistical analysis, the normality and homogeneity of variance of the data were assessed. The differences between groups were determined by one-way analysis of variance (ANOVA) followed by Tukey multiple comparison test using SPSS 17.0 software package (SPSS). When *P* < 0.05, the difference was considered significant.

## Results

### Establishment of simulated microgravity culture condition

**Fig 1:**
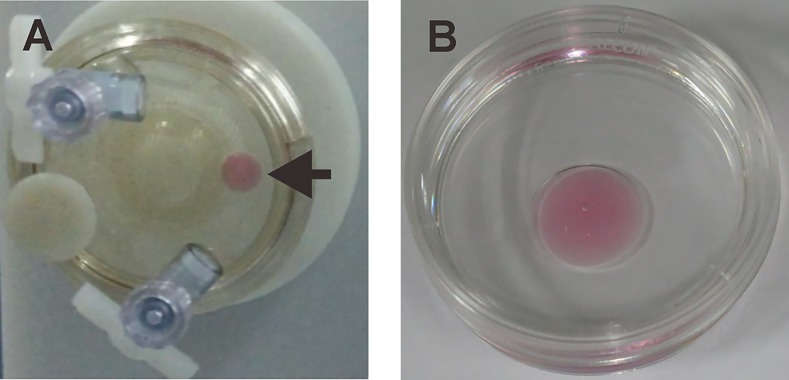
Setup of simulated microgravity and normal 1 *g* culture conditions. (A) s-μ*g* was created by the rotating wall vessel at a specific speed when a droplet of medium containing ovarian tissue or follicles was in a state of constant free-fall within the moving oil. (B) A droplet of medium containing ovarian tissue or follicles was placed on the surface of a petri dish covered with mineral oil to serve 1 *g* condition as a control. The arrow indicates a droplet of medium.

A droplet of 150 μl medium containing one piece of ovarian tissue or three encapsulated follicles was placed into the oil inside the vessel (HARV). At 25 rotations per minute, the droplet was in a state of constant free-fall within the rotating oil, mimicking some aspects of microgravity (Fig 1A). A droplet of 150 μl medium with alginate beads containing single follicles or ovarian tissue were cultured in 35×10mm dishes (BD) as a control (Fig 1B).

### Simulated microgravity compromises follicle survival in the cultured ovarian tissue

**Fig 2:**
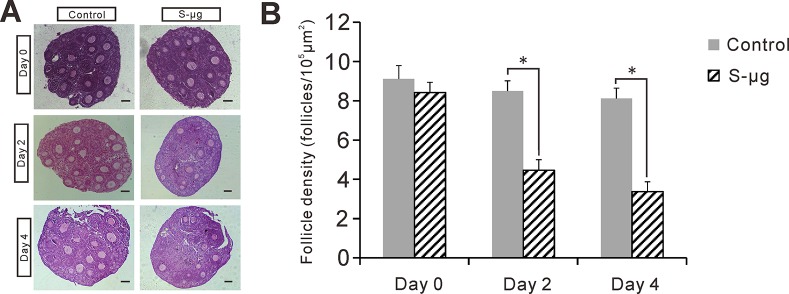
Effects of s-μ*g* on the ovarian tissue culture. (A) Representative H&E staining sections of ovarian tissue cultured under 1 *g* (control) or s-μ*g* conditions for 0, 2 and 4 days. Bar = 50 μm. (B) Evaluation of follicle density in the ovarian tissue. Error bar represents 1 SEM, *: *P* < 0.05.

**Fig 3:**
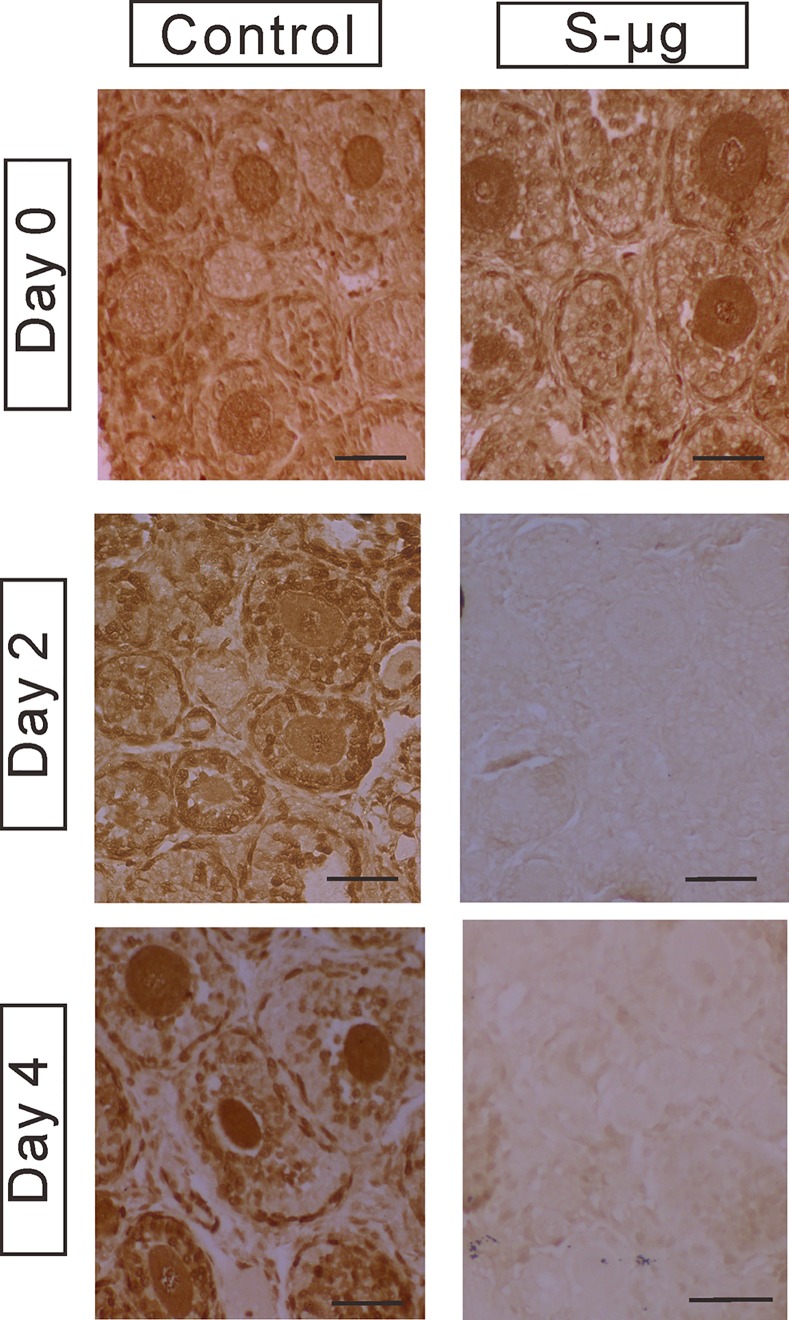
Immunohistochemistry of PCNA expression. PCNA expression in the ovarian tissue cultured under 1 *g* (control) or s-μ*g* conditions for 0, 2 and 4 days. Bar = 50 μm.

To study the effects of s-μ*g* on preantral follicle development in vitro, ovarian tissue containing preantral follicles from 14-day-old mice was cultured under conditions of 1 *g* (control) or s-μ*g* conditions. The number of health follicles was counted in the H&E stained sections in two groups after 0, 2, and 4 days of culture (Fig 2). By day 2 of culture, there were 8.5±0.5 follicles/10^5^ μm^2^ (n = 6) and 4.5±0.5 follicles/10^5^ μm^2^ (n = 6) in the control and s-μ*g* groups, respectively. By day 4, there was only 3.4±0.5 follicles/10^5^ μm^2^ in the s-μ*g* group compared with 8.1±0.5 follicles/10^5^ μm^2^ in the control group. The follicle survival in the ovarian tissue treated under s-μ*g* was significantly lower than that in the normal gravity condition even from day 2 after onset of treatment (*P*<0.05), and at the time of 4-day treatment as well (*P*<0.05). This observation was confirmed by PCNA immunohistochemistry. PCNA immunoreactivity was detected in the granulosa cells of the preantral follicles on day 0, 2, and 4 of culture in the control group (Fig 3). However, PCNA positive signals were undetectable at day 2 and 4 of the culture in the ovarian tissue under the s-μ*g* condition (Fig 3).

### Simulated microgravity compromises oocyte function *in vitro*

**Fig 4:**
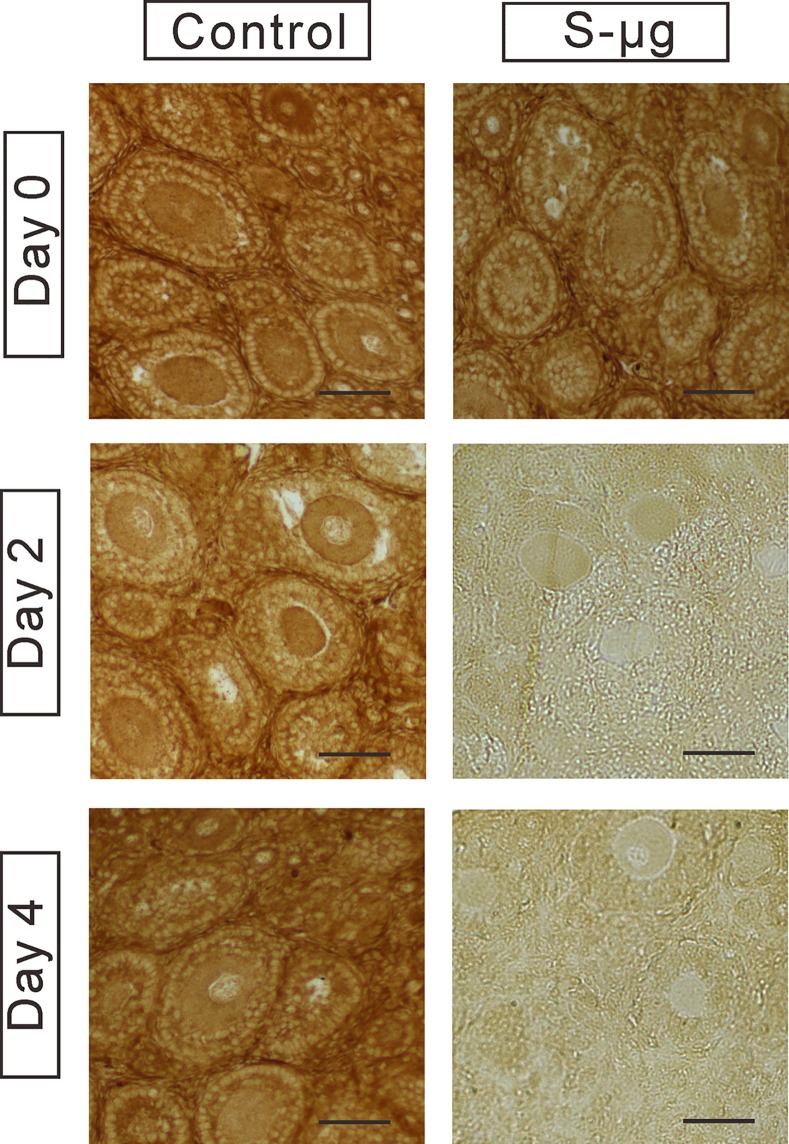
Immunohistochemistry of GDF-9 expression. GDP-9 expression in the ovarian tissue cultured under 1 *g* (control) or s-μ*g* conditions for 0, 2 and 4 days. Bar = 50 μm.

To investigate whether the decrease of the number of health preantral follicles was reflected in the oocyte functionality, the expression of the oocyte-specific marker GDF-9 was examined by immunohistochemistry. GDF-9 immunoreactive signals were detected in the preantral follicles on day 0, 2 and 4 of culture in the control group (Fig 4). Whereas GDF-9 protein expression in preantral follicles was remarkably decreased at day 2 and day 4 of culture in the ovarian tissue under the s-μ*g* condition, suggesting that simulated microgravity may be a detrimental mechanical stimulus to oocytes development.

### Simulated microgravity compromises survival of encapsulated follicles *in vitro*

**Fig 5:**
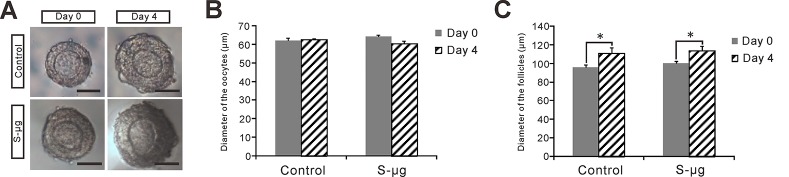
Effects of s-μ*g* on the culture of encapsulated preantral follicles. (A) Representative isolated preantral follicles cultured under 1 *g* (control) or s-μ*g* conditions for 0, and 4 days. Bar = 50 μm. (B) Follicle diameter and (C) oocyte diameter were measured and compared between control and s-μ*g* groups. Error bar represents 1 SEM, *: *P* < 0.05.

To further confirm the effects of s-μ*g* on early preantral follicle development, preantral follicle with diameter between 90–100 μm were isolated, encapsulated in alginate, and cultured under 1 *g* or s-μ*g* conditions for 4 days. 76.8%±5.3% (n = 227) and 54.4%±6.7% (n = 249) preantral follicles maintained intact 3-D structure (Fig 5A) and survived at 1 *g* and s-μ*g* condition, respectively (*P*<0.05). Cell viability assay shew that 90±8% (n = 10) and 81.5±5% (n = 10) follicles with less than 10% dead granulosa cells (S1 Fig) at 1 *g* and s-μ*g* condition, respectively (*P*<0.05). Follicle diameter and oocyte diameter were measured at the beginning of culture (day 0) and day 4 of culture. Oocyte growth was not detected from day 0 to day 4 at 1 *g* condition (62.0±1.1 μm (n = 40) and 62.2±0.9 μm (n = 49), respectively (*P*>0.05)) and at s-μ*g* condition (64.1±0.6 μm (n = 47) and 60.2±1.3 μm (n = 53), respectively (*P*>0.05)) (Fig 5B). Follicle diameter significantly increased from day 0 to day 4 at 1 *g* condition (95.8±2.7 μm (n = 40) and 110.6±6.2 μm (n = 49), respectively (*P*<0.05)) and at s-μ*g* condition (100.1±1.9 μm (n = 47) and 113.4±4.9 μm (n = 53), respectively (*P*<0.05)) (Fig 5C). Oocyte size in the follicles cultured for 4 days under two different gravities has no significant differences.

### Simulated microgravity compromises oocyte ultrastructural integrity

**Fig 6:**
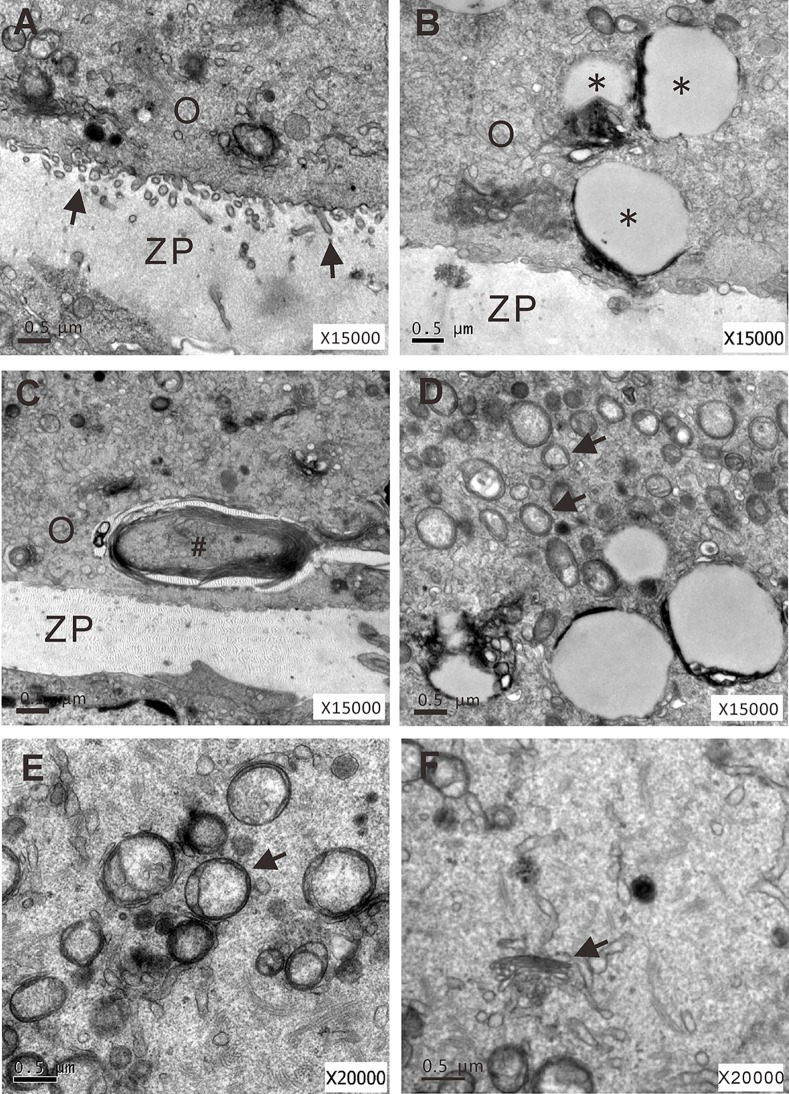
Ultrastructural analysis of isolated preantral follicles cultured under 1 *g* or s-μ*g* conditions for 4 days. (A) Oocyte cultured under 1 *g* shows microvilli (arrows) extending into the zona pellucida. (B-F) Oocytes cultured under s-μ*g* show large vacuoles (*), multilamellar bodies (#), lipid droplets (arrows), vacuolated mitochondria lacking cristae (arrow), and dispersing Golgi apparatus (arrow), respectively. O: oocyte; ZP: zona pellucida. Bar = 0.5 μm.

We further examined the ultrastructure of oocyte organelles in the encapsulated follicles at day 4 of culture at 1 *g* (n = 5) and s-μ*g* (n = 5) conditions. Oocytes under 1 *g* gravity exhibited multiple oocyte microvilli extending into the zona pellucida (Fig 6A). No oocyte microvilli, but abnormal structures, such as vacuoles (Fig 6B), multilamellar bodies (Fig 6C), lipid droplets surrounded by a double membrane (Fig 6D), vacuolated mitochondria without cristae (Fig 6E), and dispersed Golgi apparatus (Fig 6F) were observed in the oocytes cultured in the s-μ*g* vessel.

## Discussion

The development of tools like RWV provides new strategies in the field of tissue engineering [9]. Recent research reported that scaffold-free 3D culture under s-μ*g* condition, such as a rotating wall vessel, supported for tissue engineering, such as cartilage regeneration from chondrocytes, artificial vessel constructions from endothelial cells, and aggregated spheroids from thyroid cells [8]. However, it need further experimental verification whether the s-μ*g* condition can effectively promote the tissue development. In our study, the RWV apparatus equipped with HARVs provided a simulate microgravity condition for droplets of 150 μl medium containing one piece of ovarian tissue or three encapsulated follicles in the context of mineral oil at 25 rotations per minutes. The vessel spin around a horizontal axis allowed for permeation of oxygen and carbon dioxide across a membrane in the back, providing efficient oxygenation and very low shear stress [16, 17]. At the moment, studies analyzing simulated microgravity’s effects on mouse oocyte maturation and early embryo development have shown detrimental mechanisms [14, 15]. Our study indicated that simulated microgravity compromised follicle survival in the cultured ovarian tissue and isolated preantral follicles from 14-day-old mice. Other studies showed that simulated microgravity induced apoptosis in endothelial cells, gastric cells and B lymphoblasts [18–20], but not fetal fibroblasts [21], suggesting that the effects of microgravity on cell injury may be cell type-dependent.

We examined the expression of GDF-9, a member of the TGF-β superfamily, in the cultured oocytes under the s-μ*g* condition. Growth differentiation factor-9 (GDF9), an oocyte secrete factor, regulates the development of the surrounding granulosa cells in ovarian follicles [22]. GDF9 is the oocyte-specific gene product of primary and growing follicles in mouse, rat and human ovaries [23–25]. GDF9^-/-^ female mouse ovarian follicles are arrested at the primary follicle stage, indicating that GDF9 is responsible for primary follicle growth [23, 26–29]. Moreover, the expression of GDF9 had a decreased level *in vitro* than *in vivo* during the preantral stage of folliculogenesis that may correlated with the less quality of oocytes produced *in vitro* [5]. Our studies showed that simulated microgravity substantially suppressed GDF-9 expression in oocytes compared with that in the control group. We further analysed the expression of proliferating cell nuclear antigen (PCNA), as an indicator for the development of granulosa cells. PCNA is a protein involved in cell cycle regulation. The expression of PCNA in granulosa cells correlates with the initiation of folliculogenesis, and it appears in the cells only when they begin to grow [30]. Our studies showed that simulated microgravity condition suppressed PCNA expression in the granulosa cells compared with that in the control group. Once the granulosa cells were initiated to divide, the follicle diameter would increase significantly because of the proliferation of granulosa cells at the preantral stage of folliculogenesis. Whiles oocyte growth is a long-term process from preantral stage (~70μm in diameter) to the ovulatory stage (~120 μm in diameter), and had no obvious change at this stage as shown in our studies.

At ultrastructural level, microgravity caused cytoskeleton and membrane structural changes [31, 32]. In a report, simulated microgravity disturbed spindle organization and induced cytoplasmic blebbing in GV and MI oocytes [14]. In the present study, we found that oocyte microvilli withdrawn from the zona pellucida under the simulated microgravity treatment, which were similarly observed in the GDF-9-deficient follicles [26]. We also found that simulated microgravity produced large vacuoles in the periphery of the oocytes. Vacuolization is a common cytoplasmic abnormality in oocytes. The appearance of the large vacuoles may be the results of uncontrollable endocytosis or fusion of the vesicles produced by the smooth endoplasmic reticulum and Golgi apparatus. Moreover, we found that simulated microgravity treatment leaded to the appearances of numerous lipid droplets and several multiamellar bodies in the oocytes, suggesting that simulated microgravity promoted autophagy in oocytes.

Weightless condition provides a very special environment for the cells because of its lack of sedimentation for convection. For a long time, it has been clear that microgravity induces alterations in human cells [8, 33, 34]. However, the mechanisms are still unknown. Exposing cells to real microgravity is either very expensive or time limit. In vitro experiments using s-μ*g* devices can provide valuable information about modulations in gene expression profiles, signal transduction, and ultra-structures of cellular organelles in different types of cells, including oocytes, induced by altered gravity conditions. Further studies will be needed to investigate the mechanisms that cause the detrimental effects on the cells, such as oocytes, for improving space medicine and developing new treatment strategies.

## Supporting Information

S1 Fig Cell viability assay. (A): ~100% live granulosa cells (green) of a cultured follicle. (B): a follicle has live granulosa cells (green), as well as more than 10% dead cells (red).
(PDF)
