# Supplementary material for: Simulated Microgravity Using a Rotary Culture System Compromises the In Vitro Development of Mouse Preantral Follicles
Source: PLoS One. 2016 Mar 10;11(3):e0151062. doi: 10.1371/journal.pone.0151062 (PMC4786255; doi:10.1371/journal.pone.0151062)
Supplement: S1 Fig — (A): ~100% live granulosa cells (green) of a cultured follicle. (B): a follicle has live granulosa cells (green), as well as more than 10% dead cells (red). (PDF) [file pone.0151062.s001.pdf]

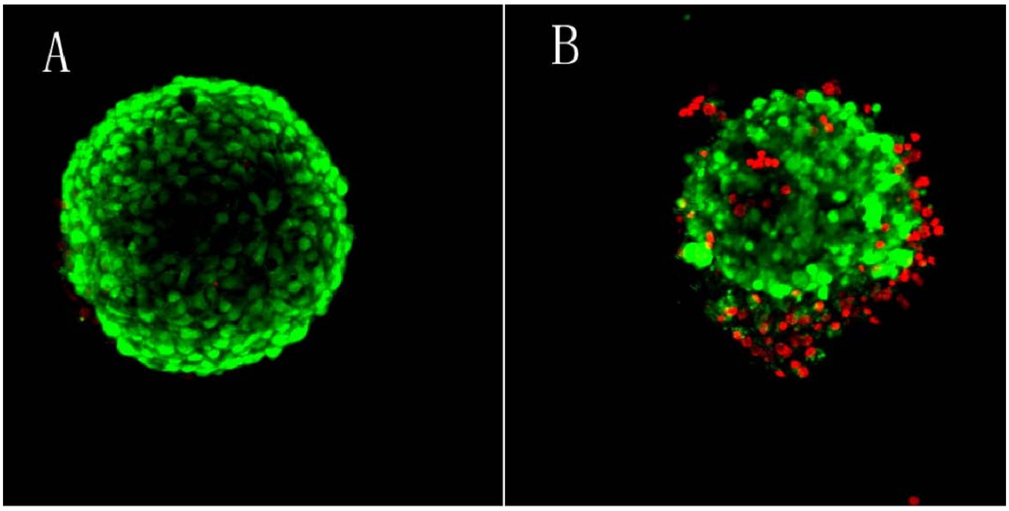

S1 Fig. Cell viability assay. (A): ~100% live granulosa cells (green) of a cultured follicle. (B): a follicle has live granulosa cells (green), as well as more than 10% dead cells (red).
